# Urocortin Expression in Endometriosis: A Systematic Review 

**DOI:** 10.22074/ijfs.2019.5488

**Published:** 2019-01-06

**Authors:** Vasilios Pergialiotis, Nikoletta Maria Tagkou, Athina Tsimpiktsioglou, Olga Klavdianou, Antonia Neonaki, Pantelis Trompoukis

**Affiliations:** 1 Laboratory of Experimental Surgery and Surgical Research N.S. Christeas, Athens, Greece; 2 Third Department of Obstetrics and Gynecology, Attikon University Hospital, National and Kapodistrian University of Athens, Athens, Greece

**Keywords:** Endometrioma, Endometriosis, Urocortin

## Abstract

Urocortin (UCN) is a neuropeptide that belongs to the corticotrophin-releasing hormone family and is expressed by eutopic and ectopic human endometria. The past years, this expression has been thoroughly investigated in the field of endometriosis. The objective of this systematic review is to accumulate current evidence related to the expression of UCN in tissue and blood samples of patients suffering from endometriosis. Literature search was designed accord- ing to the Preferred Reporting Items for Systematic Reviews and Meta-Analyses (PRISMA) guidelines and primarily conducted using the Medline (1966-2018), Scopus (2004-2018), EMBASE (1947-2018) and Clinicaltrials.gov (2008- 2018) databases, along with the reference lists of electronically retrieved full-text papers. Overall, eight studies were retrieved. Current evidence suggests that the expression of UCN is increased in patients with ovarian endometriomas and that its levels may correlate with the severity of the disease. The diagnostic efficacy of UCN1 plasma levels was evaluated in three studies. Two of them suggested that the sensitivity and specificity of the method may reach, and even exceed, 80%. However, the wide variation in outcome reporting and outcome reporting measures in endome- triosis among the included studies precludes meta-analysis of available data. Therefore, although UCN seems to be a promising biomarker for the identification and follow-up of patients that suffer from endometriosis, more studies are needed to reach firm conclusions with respect to its predictive accuracy.

## Introduction

Endometriosis is a benign inflammatory gynecological disease that manifests in women of reproductive age and is defined as the presence of stromal and viable endometrial glands outside the uterine cavity. It has an estimated prevalence of up to 10% in the general population and is associated with chronic pelvic pain, dysmenorrhea and infertility (1, 2). The pathogenic mechanisms, however, still remain unclear and the aetiology of the disease is believed to be multifactorial. Various endocrinological and immunological factors have been investigated and are considered to significantly contribute to its pathophysiology (3, 4).

During the past few years, several novel serum biomarkers have been proposed for the early diagnosis of endometriosis. The most consistently studied molecule is cancer antigen 125 (CA-125), a glycoprotein that has been established as a biomarker for the follow-up of patients with epithelial ovarian cancer. Similarly, in endometriosis, CA-125 can only be used as a prognostic rather than a diagnostic marker as it is accompanied by a significant amount of false negative results (5).

Recently, urocortin (UCN) has been extensively investigated in the field of endometriosis. UCN is a neuropeptide that belongs to the corticotrophin-releasing hormone (CRH) family and is expressed by eutopic and ectopic human endometria and is thought to play a role during decidualization (6, 7). Because of its paracrine and immunomodulatory nature, UCN is thought to contribute to the pathogenesis of endometriosis. To date, three different isoforms have been described (UCN1, UCN2 and UCN3), all of which exert their biological action by activating corticotropin-releasing hormone (CRH) receptors 1 and 2. Their main difference relies in the fact that UCN1 binds to both CRH1 and CRH2, whereas UCN2 and UCN3 bind selectively to CRH2 (8).

To date, it remains unclear whether UCN may be used as a screening and/or prognostic biomarker of endometriosis. The objective of this systematic review is to accumulate current evidence related to the expression of UCN in tissue and blood samples of patients suffering from endometriosis and provide directions for future research.

## Materials and Methods

### Study design

The present systematic review was designed according to the Preferred Reporting Items for Systematic Reviews and Meta-Analyses (PRISMA) guidelines (9). Eligibility criteria were assessed by the authors. Briefly, date and language restrictions were avoided during the literature search. All observational studies (both prospective and retrospective) that presented data relevant to the expression of UCN in tissue and blood samples of patients with endometriosis were included in this systematic review. Studies that defined their controls as either women with no pathology or women with other benign pathology were evaluated and included. Review articles, animal studies and case reports were excluded from the present review. The selection process took place in three consecutive stages. Firstly, the titles and abstracts of all electronic articles were screened to assess their eligibility. Subsequently, the articles that met or were presumed to meet the criteria were retrieved as full texts. In the final stage references of articles that were retrieved in full text were evaluated to identify studies that might have been overlooked during the electronic search. Any discrepancies in the methodology, retrieval of articles and statistical analysis were resolved through the consensus of all authors.

### Literature search and data collection

**Fig.1:**
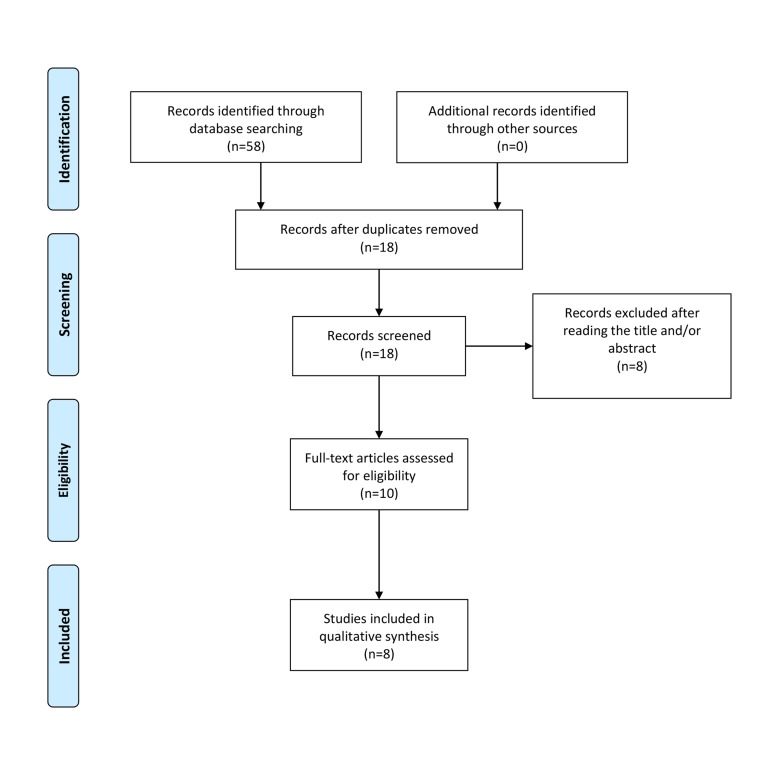
Search plot diagram.

Literature search was primarily conducted using the Medline (1966-2018), Scopus (2004-2018), EMBASE (1947-2018) and Clinicaltrials.gov (2008-2018) databases, along with the reference lists of electronically retrieved full-text papers. Additional sources were identified through the Google Scholar (2004-2018) database. Our last search was on the 04 February 2018. The search strategy included the words “endometriosis and urocortin” and is schematically presented in the PRISMA flow diagram (Fig .1).

## Results

Overall, 8 studies were included in the present systematic review and outcomes from a total of 567 women were assessed (10-18). The methodological characteristics of included studies are presented in Table 1. One study was excluded from the present systematic review as it presented preliminary data that were solely based on immunohistochemistry (13).

### *UCN, UCN2, UCN3* in utopic endometrium and endometriotic lesions

Kempuraj et al. (13) investigated in this pilot study the expression of *UCN* in biopsies from 10 patients with endometriosis and 3 patients that did not have endometriosis. Although they did not perform statistical analysis on the observed differences, they found that endometriotic lesions had increased *UCN* expression compared with healthy peritoneum and normal endometrium. Two studies evaluated *UCN* transcript expression (10, 18) and one study assessed *UCN1* and *UCN2* transcript expression in eutopic endometrium and in endometriotic lesions. While *UCN2* expression did not seem to differ between eutopic endometrium and endometriomas (16), the expression levels of *UCN* and *UCN3* in the endometriotic foci (ectopic lesions) were significantly higher compared with those in eutopic endometrium of the same women (10, 16, 18). Vergetaki et al. (18) showed an almost 3.4-fold increase in the expression levels of *UCN* transcripts when ectopic and eutopic endometrium samples were compared (0.9566 ± 0.136 a.u vs. 0.2826 ± 0.075 a.u respectively).

The extent, depth of invasion and location of endometriotic lesions were shown to be associated with *UCN* transcript levels. Specifically, Carrarelli et al. (10) observed that deep infiltrating endometriosis (DIE) is associated with higher levels of *UCN* than ovarian endometriomas (OMA) (10). 

Novembri et al. (15) studied *UCN, UCN2* and *UCN3* gene expression in eutopic endometrium of healthy women and women with endometriosis during the menstrual cycle. In women suffering from endometriosis, the expression of *UCN, UCN2* and *UCN3* transcripts was the same in the secretory and the proliferative phases, whereas in healthy women *UCN* levels differed between the two phases. Specifically, *UCN2* expression had peak values during the early proliferative phase, while *UCN3* expression was at its maximum in the secretory phase (15, 16). Both *UCN2* and *UCN3* expression levels were significantly lower in women with endometriosis when compared with healthy women (16).

### Effect of UCN on the decidualization process

Decidualization is a process of endometrial remodeling that occurs in the secretory phase of the menstrual cycle and is essential for early pregnancy. Current knowledge suggests that this process is initiated by progesterone and is mediated by various molecules such as UCN. Novembri et al. (15) showed that women with endometriosis have decreased levels of CRH and UCN, and suggested that this could negatively affect decidualization.

**Table 1:** Methodological characteristics of included studies


Author	Patient	Country	Inclusion criteria	Tissue examined	UCN form	Method of assessment

Maia et al. (14)	59 vs. 38n=97	Brazil	Consecutive list of patients that undergone laparoscopy for endometriosis	Plasma	Protein	Enzyme Immunoassay
Carrarelli et al. (10)	22 vs. 26n=48	France	Women with endometriosis^*^	OMA, DIE,endometrium	RNA, IHC	qRT-PCR
Chmaj-Wierzchowska et al. (11)	48 vs. 38n=86	Poland	Consecutive list of patients that undergone laparoscopy for endometriosis or ovarian teratoma	Plasma	Protein	ELISA
Vergetaki et al. (18)	10 vs. 16n=26	Greece	Women that undergone surgery and hysteroscopy for endometriosis	DIE, endometrium	RNA, IHC	RT-PCR
Tokmak et al. (17)	46 vs. 42n=88	Turkey	Consecutive list of patients that undergone laparoscopy for OMA vs benign cysts	Plasma	Protein	ELISA
Novembri et al. (16)	41 vs. 39n=80	Italy	Women that undergone surgery for OMA	OMA, Endometrium	RNA, IHC	RT-PCR
Novembri et al. (15)	36 vs. 26n=62	Italy	Women that undergone surgery for OMA	OMA, Endometrium	RNA, IHC	RT-PCR
Florio et al. (12)	40 vs. 40n=80	Italy	Women that undergone surgery for OMA or OMA and peritoneal endometriosis	Plasma	Protein	ELISA


UCN; Urocortin, OMA; Ovarian endometrioma, DIE; Deeply infiltrating endometriosis, ^*^; Patients with both OMA and DIE lesions were excluded, IHC; Immunohistochemistry, RT-PCR; Real-time polymerase chain reaction, and ELISA; Enzyme-linked immunosorbent assay.

### Plasma UCN as a diagnostic marker of endometriosis

Two studies evaluated preoperative plasma levels of UCN as a diagnostic factor that would help differentiate patients with endometriosis from patients with non-endometriotic, benign ovarian cysts (12, 17). Specifically, Florio et al. (12) reported that plasma UCN levels were two- fold higher in women with endometriomas (median 49 pg/mL, interquartile range 41-63 pg/mL) compared with controls (19 pg/mL, P<0.001). The receiver operating characteristic (ROC) analysis showed that UCN detected 88% of cases that had endometriomas with a specificity of 90% and an area under the curve (AUC) equal to 0.961 ± 0.021 (cut-off value 33 pg/ml). Positive and negative likelihood ratios for UCN were 8.8 and 0.14 respectively. On the contrary, Tokmak et al. (17) reported no difference in the expression of UCN between patients with endometriomas and the control group (4.8 ± 1.00 ng/ml vs. 4.5 ± 1.03 ng/ml, P=0.21). When the cut-off point was set at 4.16 ng/ml, the sensitivity of the UCN protein in detecting endometriosis was 76.2%, the specificity was 45.7% and the positive predictive value was 56.1%.

Chmaj-Wierzchowska et al. (11) compared UCN levels between patients with endometriomas and patients with mature teratomas. The expression of UCN was not significantly different between the two groups (252.37 ± 348.77 pg/ml vs. 256.03 ± 353.92 pg/ml, P=0.0727).

Maia et al. (14) studied plasma levels of UCN1 as a diagnostic biomarker of endometriosis among symptomatic patients. Compared with no-lesion patients (median 34 pg/ml, interquartile range 22-43 pg/ml), patients with endometriosis showed elevated UCN1 plasma levels (median 59 pg/ml, interquartile range 48-107 pg/ml). The ROC analysis identified plasma UCN1 concentration of 46 pg/mL as the best cut-off point to differentiate women with endometriosis from those with no lesions, with 76% sensitivity, 88% specificity and an AUC equal to 0.827. However, an optimal cut-off that would distinguish endometriosis from other benign pathology (including benign ovarian cysts, ovarian teratoma, hydrosalpinx, salpingitis, ectopic pregnancy, uterine leiomyoma and ovarian cancer) was not identified.

## Discussion

Current evidence suggests that UCN may be a promising factor for the identification and follow-up of patients that suffer from endometriosis. However, the methodological heterogeneity of these studies in terms of the reported measures precludes firm conclusions. The expression of UCN has been investigated post-transcription both at the transcription and protein levels. Three studies suggested that the expression of *UCN* transcripts is significantly higher in endometriotic lesions compared with eutopic endometrium of endometriotic women (10, 16, 18). Its correlation with the severity of the disease also implies that it might become a useful tool for the classification of endometriosis (10). On the other hand, data related to the plasma levels of the protein were conflicting. Specifically, two studies reported significant differences between patients with endometriosis and controls (12, 14), whereas another two reported that UCN levels did not differ between patients with endometriomas and patients with other benign cysts (11, 17). Maia et al. (14) suggested that the diagnostic accuracy of the expression at the protein level is promising, however, further evidence is needed to confirm these findings.

Despite the fact that our study is based on a meticulous review of current literature, certain limitations preclude definitive conclusions. Firstly, the wide variation in outcome reporting and outcome reporting measures of UCN expression in endometriosis precludes meta-analysis of current data. Furthermore, the correlation between the expression of UCN in endometriotic lesions and peripheral blood remains to be investigated. Its actual value as a minimally invasive diagnostic method therefore, remains to be elucidated. Future studies should also clarify whether UCN levels are elevated not only in endometriomas but also in non-ovarian endometriosis and in early stage disease. The presence of a biomarker with high sensitivity and specificity in these cases is particularly important for the differential diagnosis since, to date, minimally invasive methods are non-existent (19). Moreover, to standardise the measurement of UCN levels in daily clinical practice, further investigation is necessary to determine whether the peptide concentration is affected by factors including menstrual cycle, obesity, exercise, stress, diabetes mellitus and other chronic diseases (20). Accordingly, research should also focus on potential confounders that may affect the plasma levels of this protein, including diseases that may trigger chronic inflammation. To date, there is insufficient evidence to suggest that UCN acts as a mediator of inflammation, as its expression might have been an effect rather than a cause of inflammation (21). 

However, this interesting point deserves further investigation. Moreover, previous studies have also shown that UCN is produced by the liver and kidney of healthy animals (22) but evidence in humans is still lacking. Therefore, the evaluation of UCN in patients with hepatic and/ or liver dysfunction needs to be elucidated to help exclude these diseases as confounders. It would also be prudent to perform multivariate analyses to determine the impact of the various factors that were mentioned in this section potentially affecting UCN levels. Finally, cut-off values should be introduced to investigate the predictive efficacy of UCN. These cut-offs should be based on previous proposed values that were mentioned in this systematic review to evaluate consistency of results. Optimal cut-offs should also be reported to help future research in this field.

## Conclusion

Current evidence suggests that UCN may be a promising factor for diagnosis and/or prognosis of patients that suffer from endometriosis. However, available data are scarce and the findings of currently published studies remain to be validated. Specifically, future studies should examine whether plasma and tissue levels of UCN correlate in patients. Moreover, evaluation of the predictive accuracy of UCN with the use of pre-specified cut-off values, mentioned in the present systematic review, is needed to assess the reproducibility of previous findings in the field.
